# Steps towards establishing a new medical college in the Kingdom of Saudi Arabia: an insight into medical education in the Kingdom

**DOI:** 10.1186/s12909-015-0366-6

**Published:** 2015-05-06

**Authors:** Khalid A Bin Abdulrahman, Farid Saleh

**Affiliations:** 1 Departments of Family Medicine and Medical Education, College of Medicine, Al-Imam Mohammad Ibn Saud Islamic University, 7544 - Othman Bin Affan Rd, Al-Nada, Riyadh 13317-4233 Kingdom of Saudi Arabia; 2 Departments of Anatomy and Medical Education, College of Medicine, Al-Imam Mohammad Ibn Saud Islamic University, 7544 - Othman Bin Affan Rd, Al-Nada, Riyadh 13317-4233 Kingdom of Saudi Arabia

**Keywords:** Establishing a new medical college, Al-Imam Mohammad Ibn Saud Islamic University, Medical education in Saudi Arabia

## Abstract

**Background:**

The percentage of Saudi physicians practicing in the public health sector did not exceed 22.6% in 2009, and did not reach 20% in 2006. This is despite the fact that more than 80% of the Saudi population seeks health care in the public health sector. Such a low percentage of Saudi physicians is even significantly lower in the private health sector. With a fast growing population, and a low percentage of Saudi nationals practicing medicine, the need to establish new medical colleges in the Kingdom became a must. This study reflects on the steps followed in establishing the College of Medicine at Al-Imam Mohammad Ibn Saud Islamic University, and provides a comprehensive insight into medical education in the Kingdom.

**Methods:**

A sub-committee derived from the Saudi Medical Colleges Deans’ Committee was created and chaired by the founding dean of Al-Imam College of Medicine. The main goals of the sub-committee were to analyze the status of medical education in the Kingdom, and to produce an action plan to be followed when establishing a new medical college in the country.

**Results:**

The sub-committee produced a working document, which included recommendations and action plan. A medical college yet to be established should take into consideration right from the beginning both institutional and program accreditation. To achieve this goal, there are five main pillars to be planned by six main task forces. Embedded among these pillars are twenty-one domains. The analysis of the status of medical education in the Kingdom revealed some interesting observations, which are discussed in details in the manuscript.

**Conclusions:**

Establishing a new medical college should not be about just increasing the number of medical colleges as a response to the shortage of doctors. It is a lengthy “surgical operation” that requires careful and timely planning in order to anticipate and prevent any damage, and to ensure optimal outcomes. In this regard, a detailed analysis of what already exists and what needs to be done is crucial.

## Background

**Figure 1:**
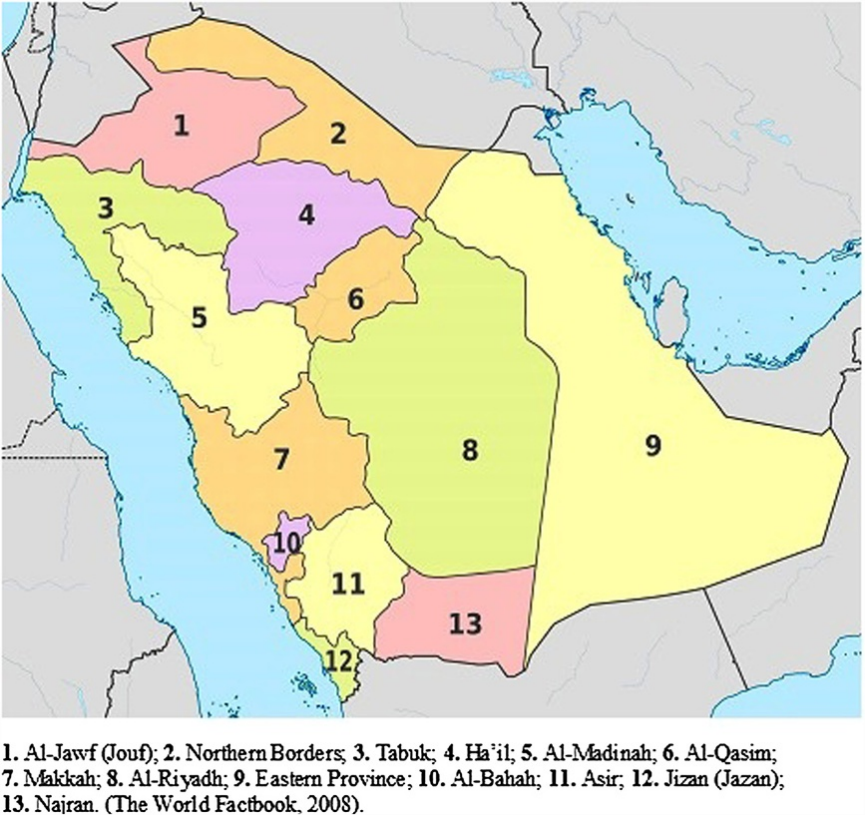
Map of Saudi Arabia showing the thirteen Provinces. Figure downloaded from Wikipedia [41].

The Kingdom of Saudi Arabia extends over an area of 2,149,690 km^2^ with a population of 26.5 million, including expatriates. It is divided into thirteen provinces, and constitutes the largest Arab state in Western Asia and the second-largest in the Arab world by land area [1] (Figure 1).

Medical education in the Kingdom has evolved since the late sixties, when the first medical college and university were established and named after the late King Saud Bin Abdulaziz. This was followed by establishing four other medical colleges, which, together with the first medical college, remained the only five colleges graduating doctors for 30 years thereafter. In 2006, the country statistics showed that the percentage of Saudi nationals holding a medical degree and practicing in the public health sector did not reach 20% [2,3]. This alarming figure necessitated the issuing of a royal decree by King Abdullah bin Abdulaziz Al-Saud, the Custodian of the Two Holy Mosques and the Chairman of the Council of Ministers and Higher Education Board, to develop and expand medical education in the Kingdom, which resulted in the establishment of new medical colleges across the country and in the opening of branches of some of the existing ones. There are currently 28 public and private medical colleges in the Kingdom, with five more public colleges on the verge to be established in the future.

The College of Medicine at Al-Imam Mohammad Bin Saud Islamic University was founded in 2007, and became operational during the academic year 2008–2009. The vision was to help in revolutionizing medical education in the Kingdom, and thus graduating medical doctors, who will become good problem-solvers, life-long learners, communicators, care-givers, decision-makers, managers, and community-leaders alerted by the changes in the community health care needs and intervening accordingly. To achieve this goal, careful and lengthy planning, testing, and retesting were followed. The aims of this study are to describe some of the steps followed in establishing the new medical college, including the preceding comprehensive analysis of medical education in the Kingdom, and to highlight some characteristic features of the program of the new college.

## Methods

This study abided by the guidelines of the Helsinki Declaration on human data. Ethical approval was granted by the College of Medicine Ethics Committee at Al-Imam University (IRB-01-2012).

Despite the urgent needs to establish new medical colleges in the Kingdom, careful considerations were made in order not to compromise the time that should be allocated to each of the steps followed while planning the establishment of the College of Medicine at Al-Imam University. In addition, the main goal that was kept in mind, and subsequently integrated in all these steps was innovation in medical education.

Soon after his appointment as a founding dean, a sub-committee derived from the Saudi Medical Colleges Deans’ Committee was created and was chaired by the founding dean. It included the Deans of three new medical colleges, namely those of Najran, Jazan, and Ha’il Universities. The main goal of the subcommittee was to review the status of medical education in the Kingdom, and to produce a list of fundamental steps to be followed by any new medical college to be established in the country.

As part of the review process, the methodology included a comprehensive analysis of all the medical colleges established in the Kingdom between the academic years 1967-1968 and 2008-2009 in relation to the main recommendations made by Flexner in his Report of 1910 for the Carnegie Foundation for the Advancement of Teaching, as well as by several international and national medical education authorities and accreditation bodies [4-29]. These recommendations are related to the foundation process, resources (costs), ratio of medical colleges per population and land area, admission and selection criteria, scientific method (curriculum), staff-to-student ratio, research, duration of the program, medical pedagogy (methods of teaching and learning), and number of graduates, to name a few. The analysis also included the local community in relation to its short-term and long-term health care needs and services [30]. The information and data obtained in the analysis were retrieved from the Saudi Ministry of Higher Education, Saudi Ministry of Health, international and national authorities on medical education, the literature, and the individual colleges’ websites for updates [2,4]. The international and national authorities on medical education included the Carnegie Foundation for the Advancement of Teaching and Carnegie Commission on Higher Education, World Federation for Medical Education, American Medical Association and Association of American Medical Colleges, Network Towards Unity for Health, Network of Community-Oriented Educational Institutions for Health Sciences, World Health Organization (WHO), Scottish Doctor Project as part of the Scottish Deans’ Medical Curriculum Group, CanMEDS as part of the Royal College of Physicians and Surgeons of Canada Phase IV Working Groups, and National Commission for Assessment and Accreditation (NCAAA) in Saudi Arabia as part of the Saudi Deans’ Committee on Medical Education [6-20]. We also referred to the AVICENNA Directory for Medicine, and the International Medical Education Directory (IMED) [21,22]. The former includes information from the survey in progress of all the world’s medical colleges, as well as information from the seventh edition of the WHO World Directory of Medical Schools, with updates submitted to the WHO Directory between 2000 and 2007 [21,23]. Other AVICENNA partners include the University of Copenhagen Faculty of Health and Medical Sciences, United Nations Educational, Scientific and Cultural Organization (UNESCO), Foundation for Advancement of International Medical Education and Research (FAIMER), Educational Commission on Foreign Medical Graduate (ECFMG), General Medical Council of the United Kingdom (GMC), Australian Medical Council (AMC), and the Institute for International Medical Education (IIME) [21,22,24-33]. Finally, we used some international medical schools as benchmarks, and these included those at Harvard, Stanford, Sydney, Toronto, Manchester, and Dundee universities.

## Results

### Action plan towards establishing a new medical college in the Kingdom of Saudi Arabia

An important cornerstone in establishing a new medical College is the appointment of a founding dean, who should have excellent medical education, leadership, management, and communication skills. These skills are essential for surveying medical education nationwide, writing unique vision, missions, goals and objectives pertaining to the new medical college, providing leadership in the implementation of these missions, goals and objectives, and acting as a catalyst for securing the resources needed for the proper accomplishment of the various tasks. In addition, the founding dean should have the expertise in planning, developing, evaluating, and implementing medical and allied health curricula [34].

**Figure 2:**
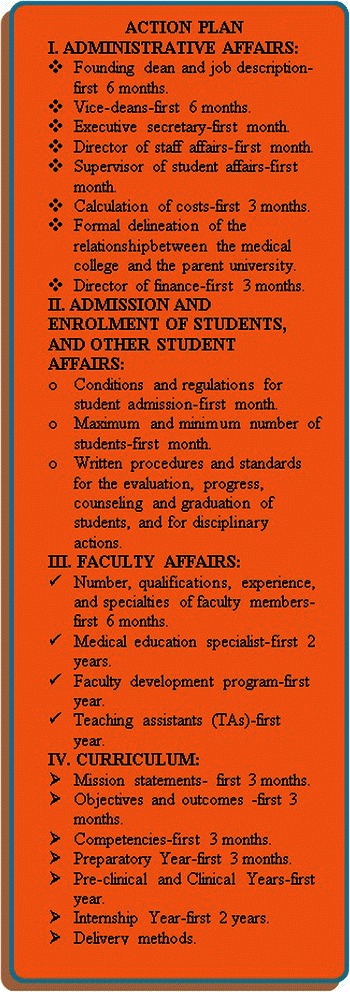
Action plan.

The recommendations made by the sub-committee included the need for the medical college yet to be established to take into consideration right from the beginning both institutional and program accreditation, which in turn should be based on international and national standards and requirements. The national standards are based on those required by NCAAA, which revolve around the standards stated by the World Federation for Medical Education (WFME). Accreditation by NCAAA is often performed at both the institution and program levels, mandatory, and should be sought within five years following the inception of a medical school and a medical program. To achieve such standards and requirements, five main pillars of a medical college should be planned by six main task forces, which in turn should be headed by the founding dean. These pillars are the setting of the institution, the medical program, the student division, the faculty division, and the resources needed. The sixth task force should act as a liaison committee, whose role is to share, feed, and get fed by the information and activities derived from the other five main task forces. Embedded among the five pillars are twenty-one domains, which include administration and governance, academic environment, and the program’s essential elements, namely curriculum management, objectives, design, content, teaching, evaluation, and the learning environment. Other domains embedded in the five pillars and pertaining to the students are their admission and selection criteria, premedical requirements, counseling, and ethical and professional conduct. Still other domains embedded in the five pillars and pertaining to the teaching staff (both faculty and teaching assistants) include their number, experience, and qualifications. The last domain pertains to the finance, space, clinical affiliations, information technology, support staff, library, and other educational resources. Planning for the domains listed above should be allocated to the six main task forces described earlier, based on the pillars to which these domains belong. The recommended action plan towards establishing a new medical college in the Kingdom, along with the suggested time frame, is summarized in Figure 2.

### Calculation of costs needed to start a new medical college in the Kingdom of Saudi Arabia

**Figure 3:**
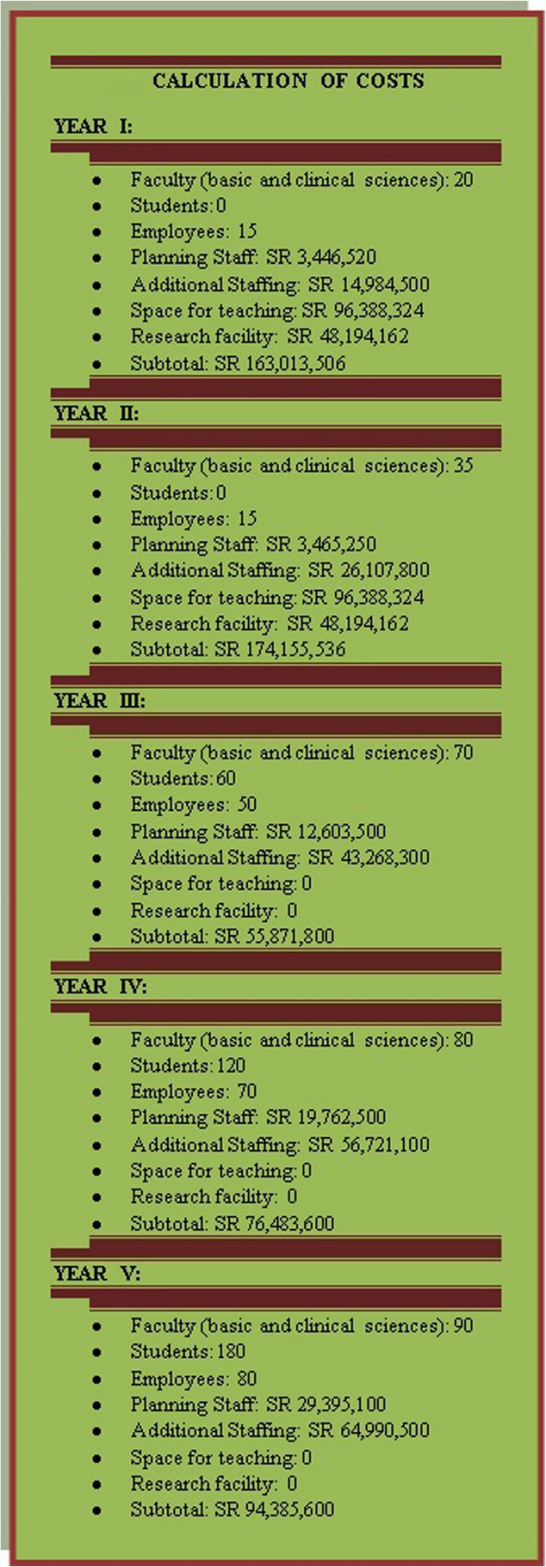
Calculation of costs.

Medical education consulting firms have estimated the costs of establishing a new medical college to range from 75 to 150 million US dollars [35,36]. We have reviewed the recommendations made by the Texas Higher Education Coordinating Board, and the requirements set by the US LCME for accrediting MD granting degree programs in relation to the estimated costs to establish a new medical college in the 21^st^ century [37]. The main revenues needed to cover six-year start-up costs for a new medical college for faculty, administration, staff, space, and research facility are summarized in Figure 3. Such calculation is based on a total first intake of sixty students, and it excludes the revenues needed to establish a university teaching hospital.

In anticipation of the establishment of the medical city, which will include the university teaching hospital, and the male and female medical colleges campuses, the College of Medicine at Al-Imam Mohammad Bin Saud Islamic University currently occupies a three-story building, which includes 25 Problem-Based Learning (PBL) rooms equipped with hexagon-shaped tables, smart boards, projectors, computers, and internet access. The building also houses a clinical skills center, dissection hall, anatomy and pathology museums, three multidisciplinary labs (equipped for histology, biochemistry, microbiology, and physiology practical sessions), seven lecture halls (two of them are interactive halls), 60 offices, a medium-size conference hall, a research center, a prayer hall, a library, students’ locker room, a cafeteria, and an underground staff parking lot.

### Comparison of the distribution of medical colleges across the five continents in relation to country population and area

We reviewed 34 countries distributed across the five continents, using the various resources listed in the Methods section. These countries included the Kingdom of Saudi Arabia, Lebanon, Iraq, Bahrain, Kuwait, Oman, Qatar, United Arab Emirates, Syria, United States, Canada, Australia, New Zealand, Japan, England, France, Germany, Italy, Netherlands, Spain, Sweden, Switzerland, Turkey, South Africa, Nigeria, Uganda, Morocco, Singapore, Malaysia, Russia, Argentina, Brazil, Colombia, India, and China. Comparison was performed between the Kingdom of Saudi Arabia and these countries in relation to ratio of medical college to country population and area.

Our results showed that the highest ratio of medical colleges per a population of one million was seen in Bahrain (2.5), followed by Lebanon (1.4), Saudi Arabia (1.1), and Malaysia (1.0), while the lowest was observed in China and Uganda (0.1). Sixty percent of the surveyed countries had a ratio ranging from 0.5 to 0.9. As for the ratio of medical college to 350,000 km^2^ of land area, the highest ratio was observed in Bahrain (1372.5), followed by Singapore (985.9) and Lebanon (200.9), while the lowest was seen in Canada (0.6). Saudi Arabia (4.6) did not differ much from the US (5.0) and Sweden (5.4). However, and except for Oman (2.3), the ratio in Saudi Arabia was less when compared to the rest of the Arabian Gulf countries (Kuwait 19.6, Qatar 30.2, and United Arab Emirates 20.9), and some South American countries (Brazil 7.4, and Colombia10.7). It was even far less when compared to some European (England 64.4, France 17.6, Germany 37.3, Italy 44.1, and Netherlands 67.4), and Asian (Japan 74.1, and Malaysia 28.6) countries.

### Comparison of the distribution of medical colleges across the thirteen provinces of the Kingdom of Saudi Arabia in relation to the province population and area, and number of Saudi practicing physicians

Comparison of the thirteen provinces in the Kingdom in relation to the distribution of medical colleges per province population revealed that the highest ratio was observed in the Northern Borders (3.1), followed by Al-Jawf (2.3) and Al-Bahah (2.2), while the lowest ratios were observed in Asir (0.5), Eastern Province (0.5), and Al-Madinah (0.7) (Table 1). On the other hand, when the provinces were compared in relation to the distribution of medical colleges per province area, the highest ratio was observed in Jazan (30), followed by Al-Bahah (23.3), Makkah (12.8), and Al-Qassim (10.8). The lowest ratio was noted in the Eastern province (1.0). The thirteen provinces also differed in relation to the ratio of Saudi practicing physicians per province population, whereby the highest ratio was observed in Makkah (56.5), followed by Al-Madinah (29) and Al-Riyadh (25). The lowest ratio was noted in the Northern Borders (2.8). When the differences among the provinces were plotted against the ratio of Saudi practicing physicians per province area, Makkah ranked first, and was followed by Jazan, Al-Riyadh, and Asir, while the Northern Borders (28.2) ranked last.

**Table 1:** **Distribution of medical schools and Saudi physicians per province population and area in the Kingdom of Saudi of Arabia (Total = 28 medical schools)**

Name of the province	Population of the province (100,000)	Area of the province (km ^ 2 ^ )	Province population density (per km ^ 2 ^ )	Number of medical schools per province	Name of the medical school(s) in the province	Ratio of medical school to province population (1,000,000)	Ratio of medical school to province area (350,000 km ^ 2 ^ )	Total number of practicing Saudi physicians (both males and females) in the province (2009)	Ratio of Saudi physicians to province population (100,000)	Ratio of Saudi physicians to province area (350,000 km ^ 2 ^ )
Al-Jawf (Jouf)	4.4	100,212	4.4	1	1. Al-Jouf.	2.3	3.5	38	8.6	132.7
Northern Borders	3.2	111,797	2.87	1	1. Northern Border (Arar).	3.1	3.1	9	2.8	28.2
Tabuk	5.6	108,000	5.19	1	1. Tabuk.	1.8	3.2	27	4.8	87.5
Ha’il	5.3	103,887	5.1	1	1. Ha’il.	1.9	3.4	28	5.3	94.3
Al-Madinah	15.1	151,990	10	1	1. Taibah.	0.7	2.3	438	29.0	1008.6
Al-Qassim	10.2	65,000	16	2	1. Qassim University. 2. Sulaiman Al-Rajhi.	2.0	10.8	97	9.5	522.3
Makkah	58	164,000	35.4	6	1. Umm Al-Qura. 2. Ta’if. 3. Batterjie. 4. Ibn Sina. 5. King Saud bin Abdulaziz for Health Sciences. 6. King Abdulaziz.	1.0	12.8	3278	56.5	6995.7
Al-Riyadh	54.6	412,000	13.2	9	1. Al-Imam Mohammad Bin Saud. 2. King Saud. 3. King Fahd Medical City. 4. King Saud bin Abdulaziz for Health Sciences. 5. Al-Faisal. 6. Al- Majma'ah. 7. Al-Maarefa. 8. Prince Salman bin Abdlaziz (Al-Kharj). 9. Princess Nora.	1.6	7.6	1363	25.0	1157.9
Eastern Province	41.2	672,522	6.1	2	1.Dammam University. 2. King Faisal (Al-Hasa).	0.5	1.0	965	23.4	502.2
Al-Bahah	4.6	15,000	30.7	1	1. Al-Bahah	2.2	23.3	42	9.1	980.0
Asir	19.1	81,000	23.6	1	1. King Khalid (Abha)	0.5	4.3	265	13.9	1145.1
Jizan (or Jazan)	13.6	11,671	117	1	1. Jazan.	0.7	30.0	169	12.4	5068.1
Najran	5	119,000	4.2	1	1. Najran.	2.0	2.9	26	5.2	76.5

### Comparison of the medical colleges in the Kingdom of Saudi Arabia in relation to some of the domains described earlier

We compared the medical colleges established in the Kingdom between the academic years 1967-1968 and 2008-2009 in relation to admission criteria, nature of the curriculum, duration of the program, faculty-to-student ratio, and number of graduates (Table 2). The results showed that the admission criteria seem to be common, but not uniformed, among the twenty-six colleges. Moreover, one of the colleges is offering a graduate medical program, in which case the GPA of the Bachelor degree and a medical college admission exam were added as additional criteria. Another common feature seen across the twenty-six colleges is that the program is divided into three phases, namely a Preparatory Year (also called Pre-Medical Phase), followed by a Pre-Clinical Phase (also called a Medical Phase) for three years and a Clinical Phase for two years. Thirty-one percent of the colleges followed the so called “traditional” curriculum, whereby the basic science courses constituted separate entities with no horizontal or vertical integration, while the rest of the colleges have adopted an integrated curriculum that is student-centered and/or outcome-based. The degree of integration seems to vary from one college to another, and it is beyond the scope of this study to describe the details of such variation. PBL was part of the curriculum in 58% of the colleges surveyed. Thirty-five percent of the colleges have integrated community medicine in their curriculum. Based on the three phases described above, the duration of the study is six years, excluding the Internship Year. An important observation was made in relation to the significantly-low full-time faculty-to-student ratio, whereby 33.3% of the colleges had a ratio of 0.1:1, 12.5% had a ratio of 0.2:1, 20.8% had a ratio of 0.3:1, 16.6% had a ratio of 0.4:1, 4.16% had a ratio of 0.6:1 and 0.7:1, 4.16% had a ratio of 2.1:1, and 4.16% had a ratio of 2.9:1. Comparison between the twenty-six medical colleges listed in Table 2, and the medical schools at Harvard, Stanford, Toronto, Manchester, Sydney, and Dundee universities revealed that these schools follow a graduate medical program lasting four years, with the exception of Manchester and Dundee whose program lasts six and five years, respectively. Moreover, the admission requirements seem to be uniformed among the North American and Australian medical schools, whereby an admission test (MCAT or GAMSAT), a GPA of the pre-medical courses taken during the undergraduate degree, and structured interviews constitute the essential criteria for admission. A striking difference noted in our comparison as well was that the full-time faculty-to-student ratio at the above listed six medical schools ranged from 1.7 to 12.6, which is significantly higher than the ratios reported in the Saudi medical colleges in this study.

**Table 2:** **Comparison of the Medical Colleges established in the Kingdom of Saudi Arabia between the academic years 1967–1968 and 2008–2009 in relation to some of the recommendations made by Flexner and other international medical education authorities and accreditation bodies**

No.	Name of the medical college, and year it became operational	Province in which the medical college/school is located	Admission criteria	Description of the program	Program duration excluding the Internship Year, and including the Preparatory Year (years)	Total student enrollment (academic year 2008-2009)		Full-time faculty (academic year 2008-2009)	Faculty-to-student ratio	Total number of graduates (academic year 2007-2008)
						New students	Existing students			
1	**King Saud University College of Medicine (1967)**	Al-Riyadh	**-**Aptitude test (30%). **-** Achievement test (40%). **-** High school exam (30%). - Preparatory Year GPA **-** Interview (Pass/Fail).	**-**Preparatory Year. -Pre-clinical phase. -Clinical phase. **-** Integrated. **-** Hybrid (lectures, PBL).	6	225	1621	643	0.3:1	313
2	**King Abdulaziz University College of Medicine (1975)**	Makkah	-National entrance examination.	**-**Preparatory Year. -Pre-clinical phase. -Clinical phase. -Partly integrated. -Lecture-based, case-based, and other student-activating learning/teaching methods.	6	16	1641	536	0.3:1	312
3	**Dammam University College of Medicine (previously King Faisal University-Dammam) (1975)**	Eastern Province	**-**High school GPA. **-**Aptitude test. **-** Achievement test. -Preparatory Year GPA ≥ 3.	**-**Preparatory Year. -Pre-clinical phase. -Clinical phase. -Lecture-based.	6	278	1139	286	0.2:1	180
4	**King Khalid University College of Medicine (Abha) (1982)**	Asir	**-**High school GPA. **-**Aptitude test. **-** Achievement test. -Preparatory Year GPA.	**-**Preparatory Year. -Pre-clinical phase. -Clinical phase. -Lecture-based.	6	214	1075	194	0.1:1	88
5	**Umm Al-Qura University College of Medicine (1996)**	Makkah	**-**Aptitude test. **-** Achievement test. **-**High school GPA. -Premedical Year GPA.	**-**Preparatory Year. -Pre-clinical phase. -Clinical phase. -Lecture-based.	6	269	1364	150	0.1:1	141
6	**Taibah University College of Medicine (2001)**	Al-Madinah	**-**Aptitude test. **-** Achievement test. **-**High school GPA ≥ 90%. -Preparatory year ≥ 2.75. -Interview.	**-**Preparatory Year. -Pre-clinical phase. -Clinical phase. -Lecture-based.	6	301	1369	249	0.1:1	52
7	**King Faisal University College of Medicine (Al-Hasa) (2001)**	Eastern Province	**-**Aptitude test. **-** Achievement test. **-**High school GPA. -Preparatory Year GPA ≥ 3.	**-**Preparatory Year. -Pre-clinical phase. -Clinical phase. -Lecture-based.	6	222	551	62	0.1:1	52
8	**Jazan University College of Medicine (2001)**	Jizan (or Jazan)	**-**High school GPA ≥ 90%. **-**Aptitude test. **-** Achievement test.	**-**Preparatory Year. -Pre-clinical phase. -Clinical phase. -Community-oriented. -Integrated body systems. Problem-based.	6	115	416	151	0.3:1	53
9	**Al-Qassim University College of Medicine (2001)**	Al-Qassim		**-**Preparatory Year. -Pre-clinical phase. -Clinical phase. - Community-oriented. - Integrated. - Hybrid (lectures, PBL).	6	128	612	106	0.1:1	114
10	**King Fahd Medical City College of Medicine (2004)**	Al-Riyadh	**-**High school GPA ≥ 90%. **-**Aptitude test **-** Achievement test. -GPA ≥3.5 in Phase I. - Interview.	-Phase I (introduction to the sciences relevant to medicine). -Phase II (system-oriented clinically-based learning). -Phase III (clinical clerkship). **-** Integrated. **-** Hybrid (lectures, PBL). -Student-centered. -Community-oriented.	6	120	200	30	0.1:1	146
11	**King Saud bin Abdulaziz University for Health Sciences (2004)**	Al-Riyadh	**-** High school direct entry (recent high school degree with both aptitude and achievement exam results). **-** A semi-structured, objective interview. **-** Graduate entry BSc (GPA ≥ 3). **-** Admission exam.	**Two tracks: Track I: -**Preparatory Year. -Pre-clinical phase. -Clinical phase. **Track II:** -Pre-clinical phase. -Clinical phase. **-** Integrated. **Both tracks: -** Hybrid (lectures, PBL). - Community-oriented, and student-centered.	4 or 6	101	469	206	0.4:1	24
12	**Ibn Sina National College for Medical Studies (2004)**	Makkah	-Results of national entrance examination.-Interview.	**-**Preparatory Year. -Pre-clinical phase. -Clinical phase. -Fully-integrated. -Emphasis on family medicine. -Problem-based, and other student-activating learning/teaching methods.	6	50	80	91	0.7:1	N/A
13	**Ta’if University College of Medicine (2005)**	Makkah	**-**High school GPA ≥ 90%. **-**Capabilities exam. **-**Cumulative exam. -Preparatory Year GPA.	**-**Preparatory Year. -Pre-clinical phase. -Clinical phase. -Spiral. -Fully-integrated. -Problem-based.	6	117	329	153	0.3:1	N/A
14	**Al-Bahah University College of Medicine (2006)**	Al-Bahah	-National entrance examination.	**-**Preparatory Year. -Pre-clinical phase. -Clinical phase. -Partly integrated. -Lecture-based, case-based, and other student-activating learning/teaching methods.	6	192	N/A	27	0.1:1	N/A
15	**Batterjee Medical College for Sciences and Technology (2007)**	Makkah	-Results of national entrance examination. -Interview.	**-**Preparatory Year. -Pre-clinical phase. -Clinical phase. -Partly-integrated. -Lecture-based.	6	251	N/A	47	0.2:1	N/A
16	**Northern Border University College of Medicine (Arar) (2007)**	Northern Borders	-Preparatory Year GPA. - Other requirements set by the college.	**-**Preparatory Year. -Pre-clinical phase. -Clinical phase. -Lecture-based.	6	62	62	28	0.2:1	N/A
17	**Tabuk University College of Medicine (2007)**	Tabuk	**-**Capabilities exam. **-**Cumulative exam. **-**High school GPA. -Premedical Year GPA.	**-**Preparatory Year. -Pre-clinical phase. -Clinical phase. -Fully integrated. -Community-based. - Lecture-based, problem-based, and other student-activating learning/teaching methods.	6	56	89	13	0.1:1	N/A
18	**Najran University College of Medicine (2007)**	Najran	**-**High school GPA ≥ 90%. **-**Capabilities exam. **-**Cumulative exam.	**-**Preparatory Year. -Pre-clinical phase. -Clinical phase. -Lecture-based.	6	30	N/A	17	0.6:1	N/A
19	**Al-Imam Mohammad Bin Saud Islamic University College of Medicine (2008)**	Al-Riyadh	**-** Preparatory Year GPA ≥ 3.5. **-** IELTS ≥ 5.	**-**Preparatory Year. - Basic Sciences (foundation of medicine & system-based). - Clerkship. **-** Curriculum is spiral, outcome-based, community-oriented, integrated, hybrid (lecture, PBL, DSL), following the SPICES model and emphasizing early clinical exposure.	6	73	N/A	20	0.3:1	N/A
20	**Al-Faisal University College of Medicine (2008)**	Al-Riyadh	**-** Entrance exam.**-** Interview.	**-**Preparatory Year. -Pre-clinical phase. -Clinical phase. **-** Integrated lecture-based, problem-based, and other student-activating learning/teaching methods.	6	50	N/A	20	0.4:1	N/A
21	**Al-Maarefa College of Medicine (2008)**	Al-Riyadh	**-**Capabilities exam score ≥ 70%. **-**Cumulative exam score ≥ 70%. **-**High school GPA ≥ 90%. IELTS ≥ 4.5. **-**College admission test.	**-**Preparatory Year. -Pre-clinical phase. -Clinical phase. -Lecture-based.	6	NR	N/A	NR	NR	N/A
22	**Al-Jawf University College of Medicine (Jouf) (2008)**	Al-Jawf (Jouf)	**-**Capabilities exam. **-**Cumulative exam. **-**High school GPA. -Premedical Year GPA.	**-**Preparatory Year. -Pre-clinical phase. -Clinical phase. -Integrated body systems. - Lecture-based.	6	65	N/A	25	0.4:1	N/A
23	**Ha’il University College of Medicine (2008)**	Ha’il	**-**Capabilities exam. **-**Cumulative exam. **-**High school GPA. -Premedical Year GPA.	**-**Preparatory Year. -Pre-clinical phase. -Clinical phase. -Integrated. -Problem-based.	6	38	N/A	77	2.1:1	N/A
24	**King Saud bin Abdulaziz University for Health Sciences (2008)**	Makkah	**-** High school direct entry (recent High school degree with both aptitude and achievement exam results. **-** A semi-structured, objective interview). **-** Graduate entry BSc (GPA ≥ 3). **-** Admission exam.	**Two tracks: Track I: -**Preparatory Year. -Pre-clinical phase. -Clinical phase. **Track II:** -Pre-clinical phase. -Clinical phase. **-** Integrated. **Both tracks: -** Hybrid (lectures, PBL). **-** Community-oriented, and student-centered.	4 or 6	64	N/A	183	2.9:1	N/A
25	**Prince Salman bin Abdulaziz University College of Medicine (Al-Kharj). (2009)**	Al-Riyadh		**-**Preparatory Year. -Pre-clinical phase. -Clinical phase. -Partly integrated. -Lecture-based, case-based, and other student-activating learning/teaching methods.	6	29	30	25	0.4:1	N/A
26	**Sulaiman Al-Rajhi College of Medicine (2009)**	Al-Qassim	- Preparatory Year at the College (GPA ≥ 3.5; TOEFEL ≥ 500). - Preparatory Year outside the College (GPA ≥ 3.75; TOEFEL ≥ 500; Entrance exam score ≥ 65%; personal interview).	**-**Preparatory Year. -Pre-clinical phase. -Clinical phase. **-** Integrated **-** Hybrid (lectures, PBL). - Community-oriented. - Acquisition of clinical skills by direct patient contact.	6	NR	N/A	NR	NR	N/A

N/A: Not applicable; NR: Not reported.

### Al-Imam Mohammad Ibn Saud Islamic University College of Medicine: the curriculum at a glance

The mission of Al-Imam College of Medicine is to prepare physicians, who would be able to meet and respond to the changing health care needs and expectations of the Saudi Arabian community [38]. The vision was to graduate medical doctors, who will become good problem-solvers, life-long learners, communicators, care-givers, decision-makers, managers, and community-leaders. Such mission and vision were always kept in mind when consulting with international experts on medical education, and while designing the curriculum of the four phases of the medical program (Table 3). These phases were based on seven main outcomes or competencies called the “Saudi Meds”, namely Doctor and Patient, Doctor and Practice, Doctor and Community, Doctor and Research, Doctor and Information Technology, Communication Skills, and Professionalism. Such competencies have been derived from the Working Paper issued by the sub-committee of the Saudi Medical Colleges Deans’ Committee, following a comprehensive review of the former of the various medical curricula around the world as well as the recommendations made by the various international authorities on medical education. Moreover, such competencies have been recommended to be uniform across all medical colleges in the Kingdom. The formative and summative measures of these seven outcomes or competencies were multiple-choice questions (MCQs), modified essay questions (MEQs), and short answers (SAs) during the Preparatory Year. In addition to these three measures, four more were included, namely students’ performance in weekly PBL sessions (twice a week), seminar presentations (three per semester), objectively-structured performance examination (OSPE), and objectively-structured clinical examination (OSCE). OSPE and OSCE are performed at the end of each body system block (duration 5-6 weeks), longitudinal course (duration 7-15 weeks), and clinical clerkship. Vertical and horizontal integration of the curriculum was ensured using the spiral approach [34]. A significant input from the Family Medicine Department across the whole curriculum was planned and executed as part of the community-oriented program. Early exposure to scientific and medical research was emphasized through the research methodology longitudinal courses, field projects, an Evidence-Based Medicine (EBM) block, and participation in research projects at various universities in North America and Australia. Mastering clinical skills was an integral part of the various body system blocks using the clinical simulation center for that purpose.

**Table 3:** **The curriculum at a glance: Al-Imam Mohammad Ibn Saud Islamic University College of Medicine**

		The Saudi Meds competencies							Outcome measures
		Doctor & Patient	Doctor & Community	Communication skills	Doctor & Research	Doctor & Information Technology	Professionalism	Doctor & Practice	(formative, and summative)
I.	Pre-medical: Preparatory Year (1 year).			x		x	x		MCQs; MEQs; SAs;
II.	Medical: Foundation of basic and clinical sciences (3 years).	x	x	x	x	x	x	x	MCQs; MEQs; SAs; PBL; Sem; OSPE; OSCE.
III.	Clerkship (2 years).	x	x	x	x	x	x	x	MCQs; MEQs; SAs; PBL; Sem; OSPE; OSCE.
IV.	Internship (1 year).	x	x	x	x	x	x	x	MCQs; MEQs; SAs; PBL; Sem; OSPE; OSCE.

MCQs: multiple-choice questions; MEQs: modified essay questions; SAs: short answers; PBL: problem-based learning; Sem: seminar; OSPE: objectively-structured performance examination; OSCE: objectively-structured clinical examination.

## Discussion

Education and health care are two very important aspects in any nation’s past, present, and future. They are both considered as essential indicators in determining how “healthy” a nation is, and whether the country is referred to as developed, developing, or underdeveloped. They are both also so much interdependent, that one could give them the analogy of “conjoined twins sharing the same heart”.

Health care continues to be a challenge across the globe. An important factor contributing to such challenge includes the ongoing increase in the world’s population, which is estimated to reach eight billion by 2050 [39]. Such population will be characterized by an aging community on one hand, and a pool of 32% of young people below 20 years of age, on the other. Other factors include world economy crisis leading to a significant decrease in the available resources, fast spread of diseases across the five continents due to mass air travel, growing resistance to antibiotics, and deficiency in the supply of medical and allied health practitioners, to name a few. Medical practitioners constitute an important element in health care provision, and the quality of their practice significantly depends on proper undergraduate and postgraduate medical education and training. Such education starts in medical schools, and is expected to continue throughout the physician’s life until he/she retires.

Medical education has evolved over the years from the so called “traditional” curriculum to “modern” curriculum. The former has been criticized for being teacher-centered, knowledge-giving, discipline-led, hospital-oriented, opportunistic (apprenticeship) and consisting of a standard program, while the latter has been advocated for being student-centered, problem-based, integrated, community-oriented, systematic, and consisting of a core program and electives. The kingdom of Saudi Arabia has witnessed a significant change over the past two decades in both the number of medical colleges available, and the nature of the curriculum these colleges follow. The College of Medicine at Al-Imam Mohammad Ibn Saud Islamic University was established in 2008, as part of the country’s health care reform needed to address the serious shortage of Saudi medical practitioners across the thirteen provinces that constitute the Kingdom.

In this study, we described the essential steps and action plan needed to establish a new medical college in the Kingdom, including the time frame and cost for each step, based on an initial intake of sixty students. As part of the planning process of establishing our medical college, a detailed comparative analysis was performed to examine the status of medical education in the Kingdom between the academic years 1967-1968 and 2008-2009 in relation to some of the recommendations made by Abraham Flexner early in the past century, as well as by international and national authorities and accrediting bodies in medical education and training. Our analysis revealed the following observations:

First, Saudi Arabia ranked third in relation to the ratio of medical schools to a population of one million, when compared with thirty-four countries across the globe, including North America, Europe, and Australia. Our review of the medical schools around the world revealed that there is no typical pattern defining the optimal number of medical colleges per population size. We support the argument that the optimal number of medical colleges should not only depend on the operating health care system and population size, but also on the population density and the need to comprehensively cover rural and urban geographical areas. Accordingly, there could be justifications for deviations from a general consensus, which could be estimated to be around one medical college per 1.5–2 million inhabitants [40]. Moreover, the Carnegie Commission on Higher Education recommended that every community with a population of at least 350,000 should be the site of a university hospital by virtue of the impact the latter would have on the local and regional community health care needs [6].

Second, the Kingdom ranked 26^th^ in relation to the ratio of medical schools to 350,000 km^2^ of land area. Although the Kingdom’s ratio seems to be close to that of the United States, yet it is significantly less than the ratios observed in Europe and the Middle East. This should necessitate re-thinking in relation to the future distribution of medical colleges in the Kingdom especially that the country has been suffering from a significant shortage in the number of Saudi practicing physicians.

Third, the intake of students and the number of graduates across the twenty-seven medical colleges included in our analysis are significantly less than what is required to compensate for the significant shortage in the number of Saudi physicians. In fact, and based on some preliminary figures for the year 2009, such as population size, population growth rate, fertility rate, total number of existing medical colleges, total number of medical graduates in the Kingdom, total number of Saudi physicians sent overseas for a fellowship, and number of Saudi physicians practicing in the public health sector, it could take around 25 years to reach a ratio of 254 publically-practicing Saudi physicians to 100,000 population. This again provides further justification in relation to the need to expand the existing medical colleges to increase their intake of students, and to establish new ones. Having said that, it is equally important to ensure that the quality of medical education should not be compromised at any stage during this process.

Fourth, the full time faculty-to-student ratio seems to be significantly less than the optimal ratio required for curricula that emphasize the student-centered approach, especially that the students being admitted to the medical colleges across the Kingdom are graduates from a 12-year-schooling system that relies heavily on didactic teaching. It is recommended that the medical colleges in the Kingdom should seriously increase the number of faculty, not only to meet the international standards for accreditation, but also to meet the significant increase in the student intake if the plan that we recommended to expand the existing medical colleges and to establish new ones was put into action in order to reach the ratio of 254 Saudi physicians per 100,000 population.

**Figure 4:**
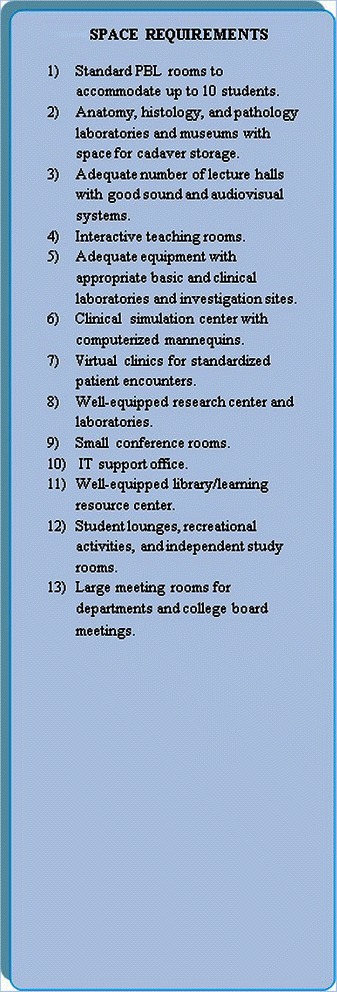
Space requirements.

Fifth, the administrative and instructional space required should be directly-proportional to the nature of the medical program to be delivered. A summary of the space requirements is presented in Figure 4.

In conclusion, this study highlighted the important steps to be followed in establishing a new medical college in the Kingdom of Saudi Arabia, based on our experience at Al-Imam Mohammad Ibn Saud Islamic University College of Medicine. As part of the planning process for establishing our College, a comprehensive analysis was performed regarding the status of medical education in the Kingdom. According to our knowledge this is the first comprehensive study of its kind in the Kingdom. Among the key lessons to be learned from this experience are that proper planning, setting up and meeting deadlines, testing and re-testing, involving the community and future students and faculty, and pulling out the right resources are essential ingredients for a “successful recipe”. Moreover, the new medical college should have a significant and direct impact on the health care needs of the community and region where the college would be located, increase the supply of well qualified physicians who are compassionate and who will be inclined to practice in the community and region, and enhance the academic standing of the university to which it belongs.
